# Plasticity-mediated collapse and recrystallization in hollow copper nanowires: a molecular dynamics simulation

**DOI:** 10.3762/bjnano.7.21

**Published:** 2016-02-10

**Authors:** Amlan Dutta, Arup Kumar Raychaudhuri, Tanusri Saha-Dasgupta

**Affiliations:** 1 Department of Condensed Matter Physics and Material Sciences, S. N. Bose National Centre for Basic Sciences, Salt Lake, Kolkata 700 098, India; 2 Unit for Nanoscience and Technology, Department of Condensed Matter Physics and Material Sciences, S. N. Bose National Centre for Basic Sciences, Salt Lake, Kolkata 700 098, India; 3 Thematic Unit of Excellence on Computational Materials Science, S. N. Bose National Centre for Basic Sciences, Salt Lake, Kolkata 700 098, India

**Keywords:** dislocations, molecular dynamics, nanowire, thermal stability

## Abstract

We study the thermal stability of hollow copper nanowires using molecular dynamics simulation. We find that the plasticity-mediated structural evolution leads to transformation of the initial hollow structure to a solid wire. The process involves three distinct stages, namely, collapse, recrystallization and slow recovery. We calculate the time scales associated with different stages of the evolution process. Our findings suggest a plasticity-mediated mechanism of collapse and recrystallization. This contradicts the prevailing notion of diffusion driven transport of vacancies from the interior to outer surface being responsible for collapse, which would involve much longer time scales as compared to the plasticity-based mechanism.

## Introduction

Nanomaterials, as compared to bulk, are associated with large surfaces and interfaces with respect to their volume. The energy corresponding to the surface of the nanomaterial is typically much larger than that of the interior. This causes an inherent structural instability with the aim to minimize the energy. Although the nanosystem can remain trapped in the surface-dominated high-energy structure during the synthesis process, it relaxes to a lower-energy structure upon annealing. This structural transition often causes significant change in the morphology of the nanomaterial. There are several such examples in literature. For instance, the rapid growth of grains in nanocrystalline metal is driven by the thermodynamic tendency of reducing the grain boundary to grain volume ratio [1–2]. Another example is that of the nanoparticles exhibiting the propensity of sintering to reduce the free surface through enhanced area of inter-particle contact [3–4]. Perhaps the most interesting example is that of Rayleigh-like instabilities exhibiting modulated patterns on the surfaces of pre-molten nanowires [5–6].

In this context, it is a pertinent question to ask what happens in case of a nanomaterial with a hollow interior. The intrinsic structural instability, as discussed above, is expected to be enhanced in such case by the presence of an additional surface. Hollow nanostructures are being used industrially as fillers for the manufacturing of lightweight composite materials. Besides this structural function, they have also been demonstrated to function as the active elements of recoverable catalysts and highly sensitive sensors [7]. Hollow nanomaterials may be created deliberately [8–10]. They can also be synthesized in an uncontrolled growth process such as flame combustion [11]. Thermal stability of hollow nanostructures has been widely discussed in literature [9]. The general understanding is that a nanostructure with hollow core is thermodynamically unstable due to the internal free surface, and shows the tendency to collapse. The kinetics of the collapse has been assumed to proceed through the slow diffusive route, where the migration of vacancies from the hollow core to the outer surface is considered to be the dominating mechanism. Accordingly, analytical theories and Monte Carlo simulations based on the process of thermally activated diffusion process have been developed and carried out [12]. However, the proposed mechanism of collapse via migration of vacancies from the inner core to the outer surface still needs to be probed through rigorous atomistic simulation without relying on the preconceived notion of diffusive transport of vacancies.

In this article, we study the above-mentioned issue by performing molecular dynamics (MD) simulation of ultra-thin single crystalline copper nanowire (NW) with hollow core. Hollow Cu nanowires have been fabricated experimentally [13–14], and studied by means of simulations [15–17]. Our study of high temperature stability of these nanowires shows interesting results. We find that the wire collapses almost immediately within a rather short span of time, thereby discarding the proposed mechanism of a slow diffusive route of vacancy migration. On the other hand, the collapse is found to proceed through creation of disordered atoms and plastic slips. Upon collapse, the hollow nanowire becomes partially amorphous, which heals through the recrystallization of disordered atoms and removal of stacking faults. Thus, the hollow nanowire transforms into a single crystalline solid nanowire having a reduced outer diameter as compared to that of the initial structure. The present study provides an atomistic description of the thermal stability of hollow nanowires, which will be useful in designing of technologically important nanomaterials with hollow cores.

## Simulation scheme

The simulated nanowire is constructed by filling up a cylindrical region with atoms in face-centered cubic (fcc) structure of copper oriented in the <111> direction. The core of the wire is made hollow by removing all the atoms within an inner cylindrical region. The results presented in the following are obtained for the hollow nanowires of 6 nm outer and 3 nm inner diameters. Calculations are carried out by varying both the inner and outer diameters, which are found to yield qualitatively similar results. The interatomic interaction is modeled using the embedded atom method (EAM) potential [18], the parameters of which have been developed by Zhou and co-workers [19]. This potential is capable of reproducing many fundamental properties of the metal including the features of major interest in the present study, e.g., stacking fault energy, vacancy formation energy, elastic constants and melting point. The hollow wire is first optimized to a minimum-energy structure and then exposed to a constant temperature maintained by means of the Nosé–Hoover thermostat [20–21]. The simulation cell in the axial direction of the nanowire is taken as about 86 nm long and periodic boundary condition is imposed. The technique of common neighbor analysis [22] is used to identify the crystal defects in the wire. All the simulations reported in the present study are performed using the MD code as implemented in the large-scale atomic/molecular massively parallel simulator (LAMMPS) developed at the Sandia National Laboratory [23], while the OVITO [24] visualization tool is employed to view the atomic configuration of the nanowire.

## Results and Discussion

### Morphological evolution

We start with the discussion of the morphological evolution, which happens during the simulation. As evidenced, the time evolution of the morphological change of hollow nanowire involves three distinct stages: (i) rapid collapse, (ii) recrystallization and (iii) slow recovery. In the following, we describe each of these stages considering the representative case at a temperature of 700 K, which is about 150 K below the melting point of the studied wire as computed through MD simulation [6].

#### Stage 1

**Figure 1:**
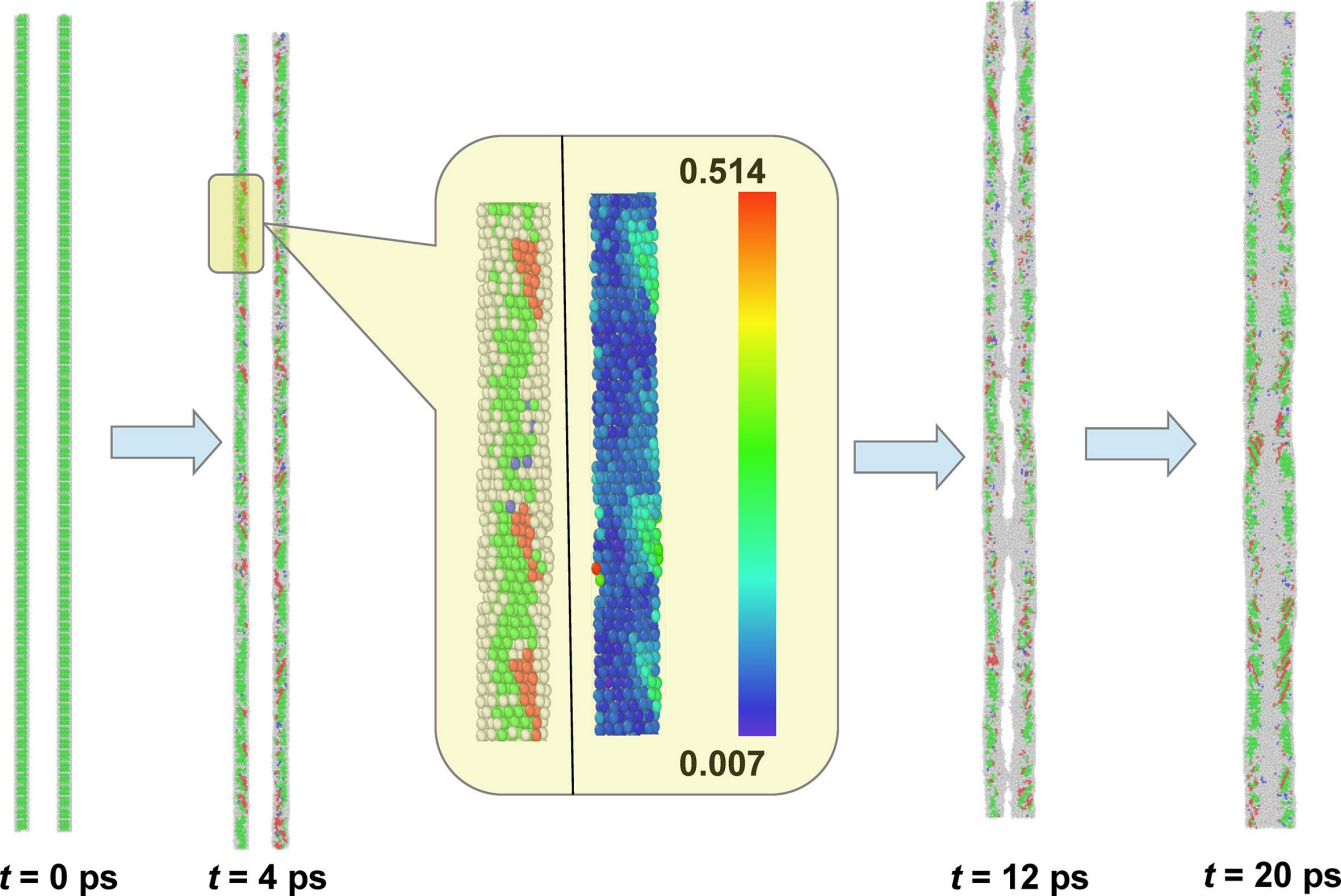
Simulation snapshots at 700 K, taken at different points in time (0, 4, 12 and 20 ps) spanning the duration of the rapid collapse. The atoms are colored according to their local structure, obtained by common neighbor analysis. Green, red and grey colors represent atoms with fcc, stacking fault and disordered configurations, respectively. The inset shows the zoomed view of the snapshot at *t* = 4 ps (left) as well as the color-coded deviatoric-strain map (right). The color bar is shown by the side. The zoomed view clearly shows the nucleation of partial dislocations at the inner wall of the hollow nanowire.

Figure 1 shows the initial stage of the simulation. The hollow structure shows an almost immediate collapse, within the first tens of picoseconds of the simulation run. At the initiation of the simulation, a large fraction of atoms in vicinity of the inner surface becomes disordered (shown as gray atoms in Figure 1), with residual crystalline atoms situated at the outer periphery of the wire. The mobility of these disordered atoms is much higher as compared to their crystalline counterparts since they access a relatively larger free volume. This causes a radially inward drift of the inner surface driven by surface tension. This inward drift results into large local stresses on the residual crystalline parts of the system. The resolved shear load can thereby exceed the critical limit for producing the Shockley dislocations. The calculated shear-strain map indicates that the partial dislocations nucleate at the inner surface and propagate towards the outer periphery, thereby leaving behind trails of stacking faults (shown as red atoms in Figure 1). This firmly establishes that instead of the conventional notion of slow and diffusive mode of collapse by vacancy migration, the collapse happens through a plasticity mediated mechanism involving the rapid drift of disordered atoms and creation of stacking faults on close-packed planes of the remaining crystalline region. At the end of this stage, the initial hollow interior gets filled by disordered atoms, surrounded by the residual crystalline atoms in the FCC structure.

#### Stage 2

**Figure 2:**
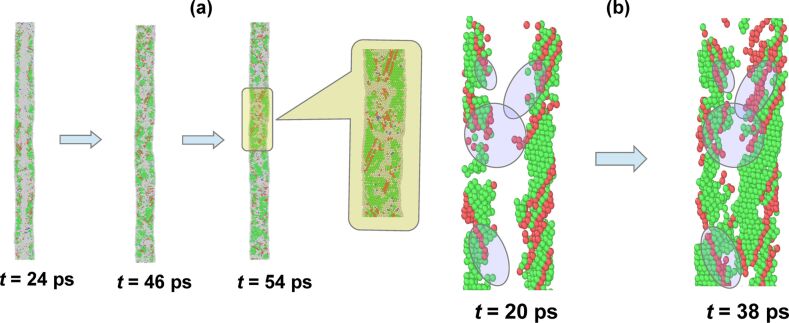
(a) Simulation snapshots at 700 K, taken at different points in time (24, 46 and 54 ps) spanning the duration of recrystallization. The atoms are colored as described in Figure 1. The inset shows the zoomed view of the snapshot at *t* = 54 ps. It is evident that most of the disordered atoms are accumulated at the termination sites of the stacking faults. (b) The stacking-fault atoms at two different instances (20 and 38 ps) of recrystallization. The stacking faults are seen to grow in the inward direction. These growth sites are highlighted by the encircled regions.

The next stage of evolution involves recrystallization of the disordered atoms. In this process, the residual crystalline parts of the collapsed structure act as nucleation centers for converting back the disordered atoms into fcc structure. Subsequently, the recrystallized part of the wire grows in the radially inward direction as see in Figure 2a, which results in the simultaneous growth of the stacking faults at the interface between the crystalline regions and the disordered atoms (cf. highlighted parts in Figure 2b). In comparison to the defect-free parts of the NW, recrystallization becomes more difficult at the boundaries of the stacking faults. Recrystallization at such a site would create a [112]/6-type dislocation and, thus, would add to the free-energy of the system. That is why most of the remaining disordered atoms are found at the terminating sites of the stacking-faults (cf. Figure 2a).

#### Stage 3

**Figure 3:**
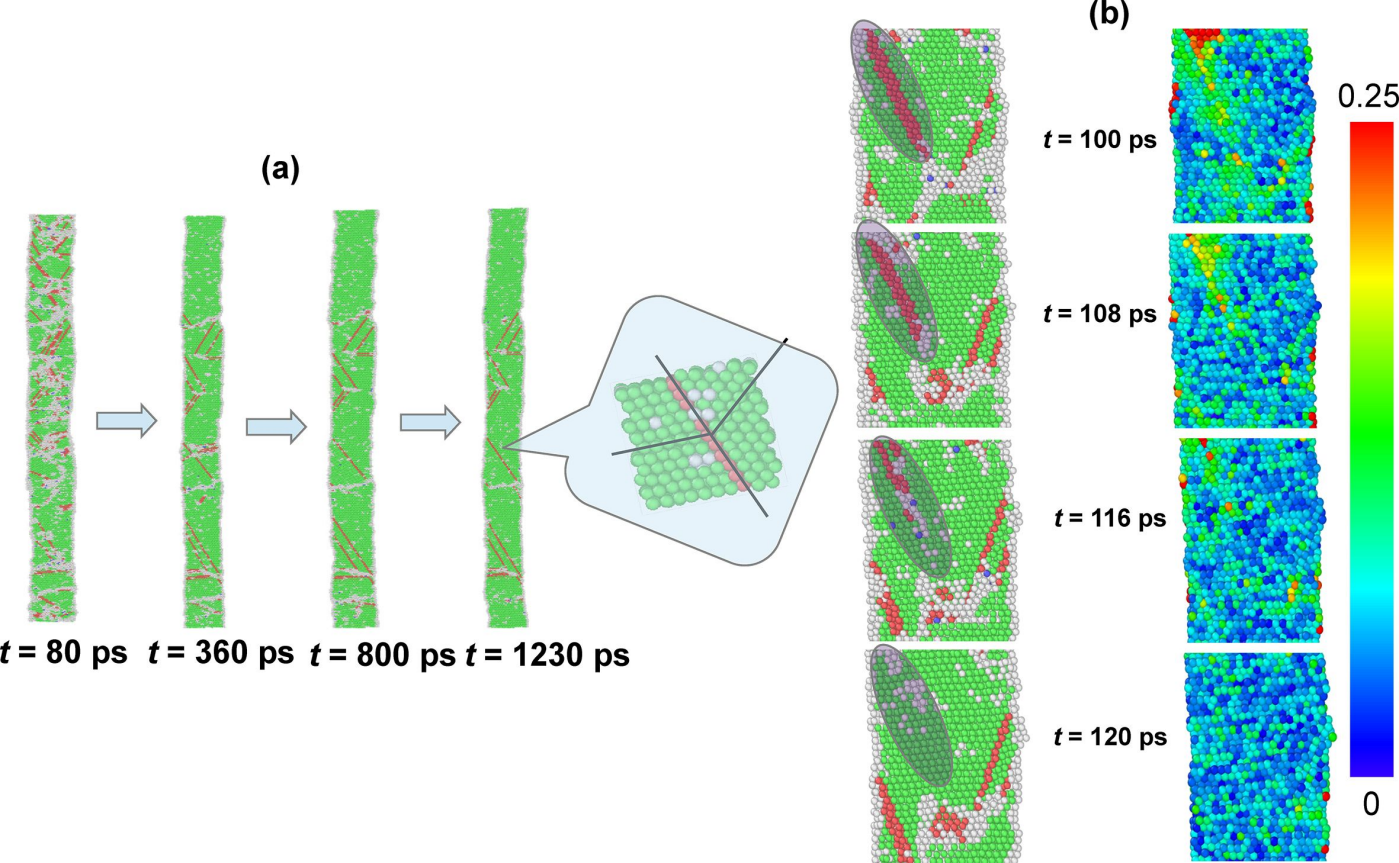
(a) Simulation snapshots at 700 K, taken at different points in time (80, 360, 800 and 1230 ps) spanning the duration of slow recovery. The final morphology is that of a single crystalline nanowire containing a few planar defects. As displayed in the zoomed view, these planar defects are the twin boundaries. (b) Zoomed view of the simulation snapshots, taken between 100 and 120 ps, highlighting the mechanism of recovery. As highlighted, the partial dislocation at the boundary of an abruptly terminated stacking fault moves towards the outer surface and finally disappears during the recovery. Color-coded snapshots indicating the atomic shear strains are also given by the side.

The third and final stage of the morphological evolution consists of disappearance of the residual disorder and stacking faults within the NW. This process is much slower as compared to that of the previous two stages. Healing of the nanostructure at this stage involves elimination of stacking faults, which is an activated process, thus exhibiting the observed slow rate. Most of the stacking faults get eliminated at this stage, and the final structure consists of only a few twin boundaries as shown in the inset of Figure 3a. As already mentioned, recrystallization of the residual disordered atoms prompts the formation of [112]/6-type partial dislocations at the boundaries of stacking faults. These partial dislocations, owing to their proximity to the outer free surface, feel image forces [25] driving them towards the surface. As a consequence, they move towards the free surface, which leads to a shrinkage of the stacking fault regions. This mechanism of recovery is demonstrated in Figure 3b. The accompanying strain map shows that the shrinkage of the stacking fault reduces the elasto-plastic strain, resulting in a structure with significantly reduced density of defects in comparison to stage 2.

**Figure 4:**
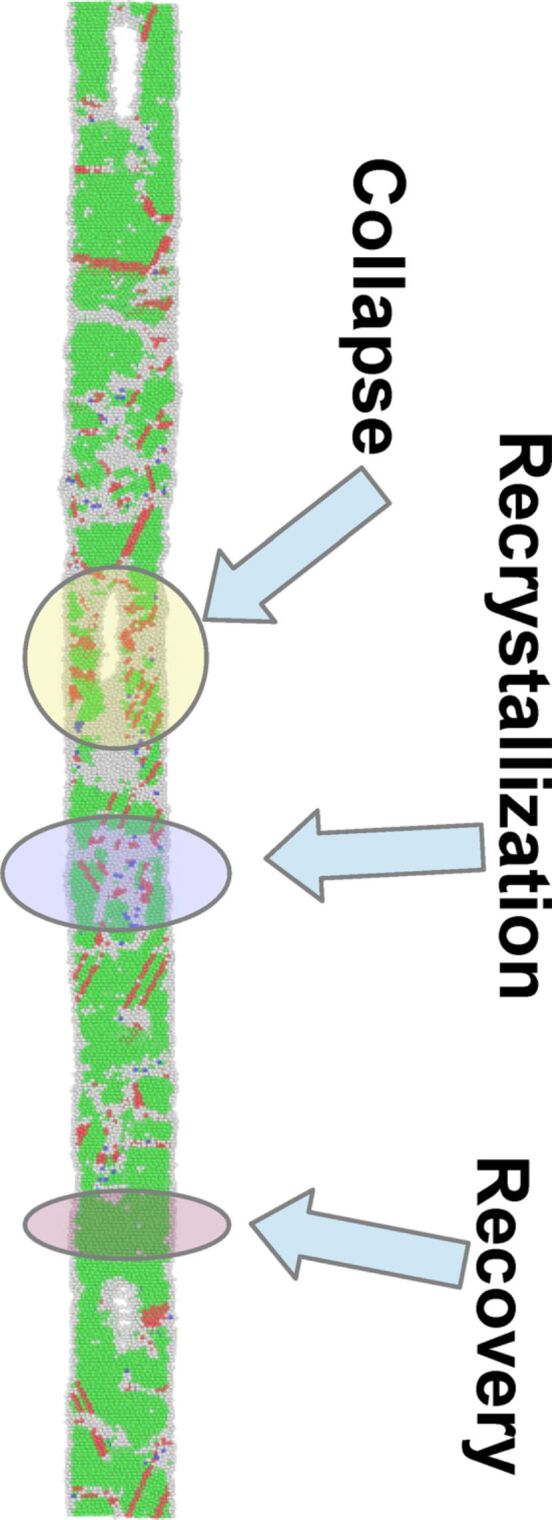
Snapshot of the nanostructure at 575 K temperature taken at *t* = 68 ps. As the snapshot shows, at this temperature, different parts of the nanowire undergo different stages of morphological evolution at the same time, namely, collapse (yellow colored highlight), recrystallization (violet colored highlight) and recovery (magenta colored highlight) .

Understandably, temperature plays a deciding role in the kinetics of the above mentioned processes. In the above described representative case at 700 K, different stages of evolution, namely collapse and subsequent recrystallization happen uniformly along the length of the nanostructure. However, this scenario is found to change as the temperature is reduced below 600 K, exhibiting qualitatively different behavior within the simulation time of 1200 ps. In the following, we refer the temperature of 600 K as the cross-over temperature, *T*_cross_, marking a crossover from the spatially non-uniform to uniform evolution. At temperatures below 600 K, some parts of the nanowire collapse earlier than the other parts. Consequently, these parts also get recrystallized before the other parts. As a result, along the length of the NW, all the three stages occur simultaneously with different parts of the wire exhibiting different stages of the evolution process, as demonstrated in Figure 4 for the wire at 575 K.

### Kinetics

Having gathered a qualitative picture of the various mechanisms underlying the different stages of morphological evolution of the hollow NW, we proceed to quantify its kinetics through quantities such as time evolution of the potential energy, radius of gyration and the atomic volume.

### Evolution of potential energy

**Figure 5:**
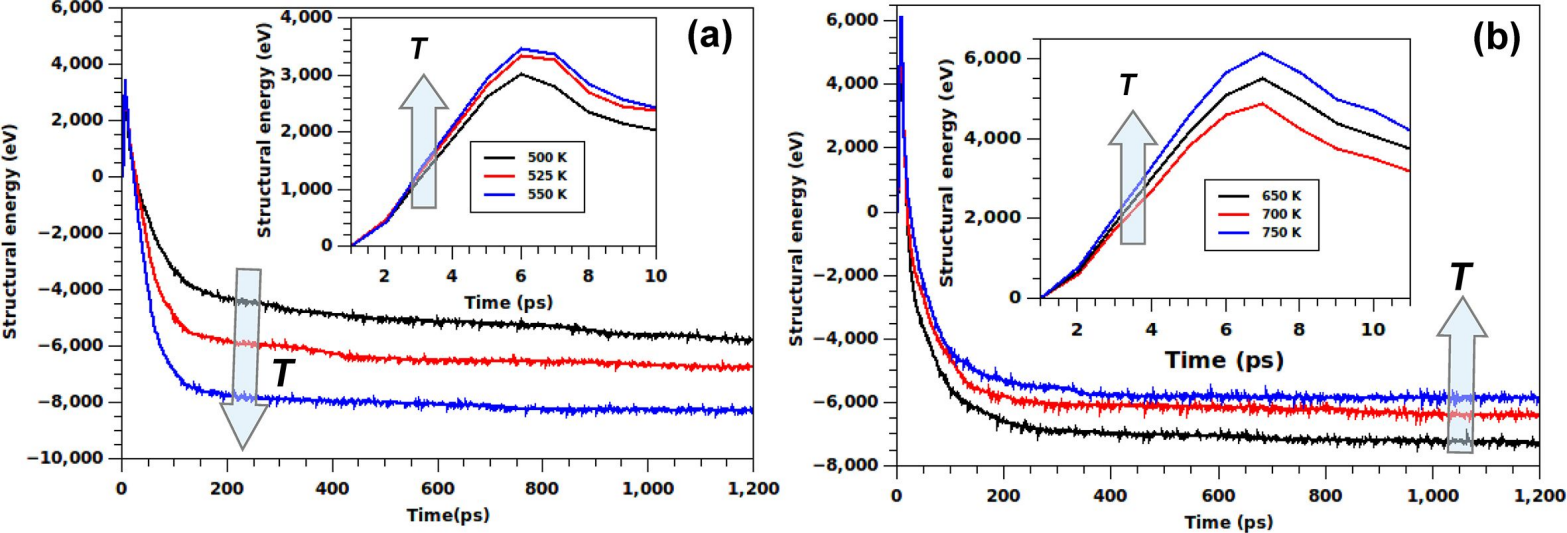
The potential energy of the simulated nanostructures as a function of the time at (a) low-temperature (*T* < *T*_cross_) and (b) high-temperature (*T* > *T*_cross_) regimes. Shown are the plots for a few representative temperatures, 500 K (black), 525 K (red), 550 K (blue) in the low-temperature regime, and 650 K (black), 700 K (red) and 750 K (blue) in the high temperature regime. The temperature-dependent trend is found to be generally different in the two regimes. The insets offer a closer look during the first few picoseconds of the simulations. Interestingly, in this short timescale, the trends are found to be qualitatively similar between the two regimes.

The potential energy of a NW, which depends on the instantaneous atomic structure, provides valuable insights regarding the kinetics of the underlying mechanism. Figure 5 shows the variation of the potential energy of the NW during the entire evolution process for temperatures above and below *T*_cross_. Qualitatively different behavior is observed in these two temperature regimes. In both cases, the structural energy initially increases with time due to thermally activated formation of disordered atoms. The potential energy reaches its peak value in the first 6–7 ps of the simulation.

Following this rise, the potential energy decreases with time, finally reaching saturation. The trends in the temperature dependence of this saturated value are found to be opposite in temperature regimes above and below *T_cross_*. In the low-temperature regime (*T* < *T*_cross_) represented in Figure 5a, the saturation value of the structural energy is found to decrease with a rise in temperature, while an opposite trend is obtained for the high-temperature regime (*T* > *T*_cross_, Figure 5b). The behavior of the saturation energy in high-temperature regime can be justified from the fact that the final structure at the end of slow recovery consists of twin boundaries only. The number of such twin boundary defects increases with the annealing temperature, thereby increasing the value of the final structural energy. On the other hand, at temperature below *T*_cross_, the final structure bears the signature of the incomplete collapse with a fraction of the free inner surface being present in the partially collapsed hollow NW, as already demonstrated in Figure 4. The extent of this residual inner surface increases upon decreasing the annealing temperature. Therefore, lowering the temperature causes a larger contribution to the potential energy, explaining the trend opposite to that of the high temperature regime.

### Radius of gyration

**Figure 6:**
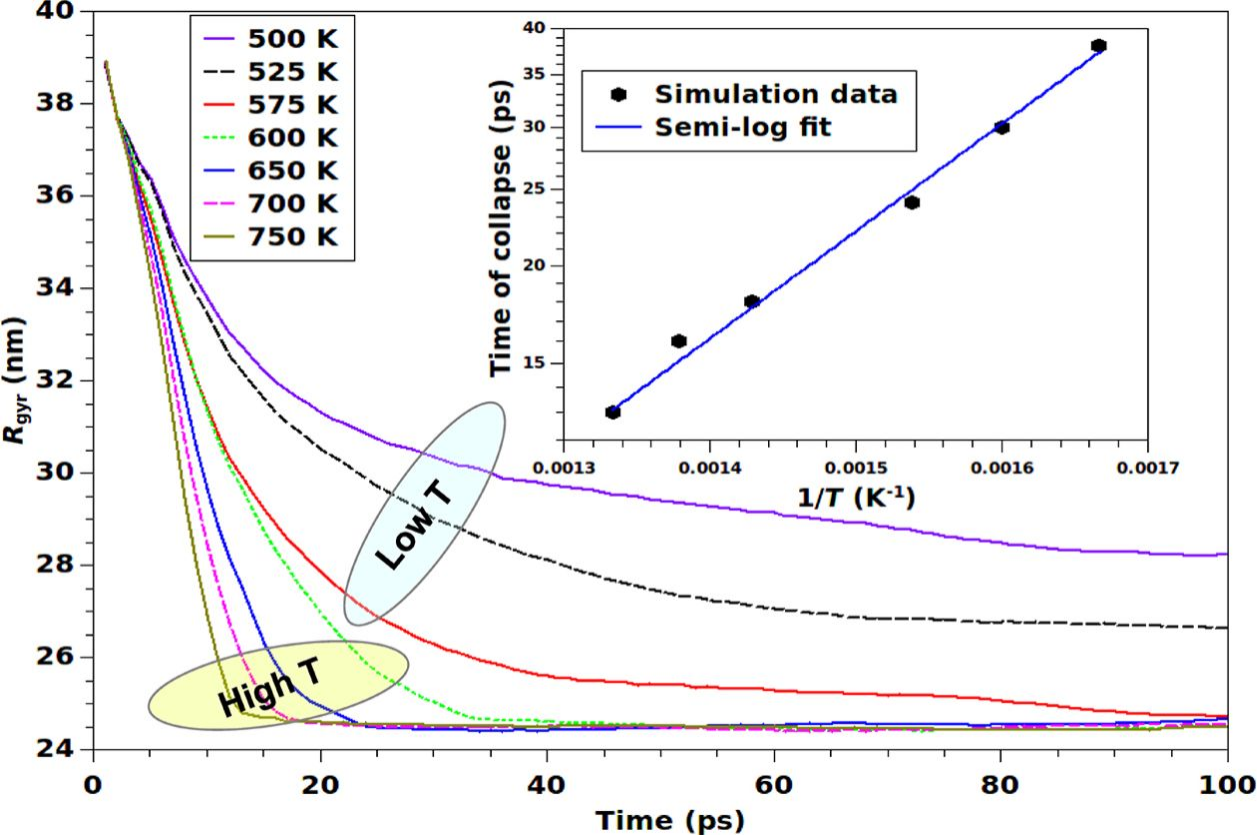
Radius of gyration plotted as a function of the simulation time for several temperatures belonging to both low-temperature and high-temperature regimes. The two temperature regimes are highlighted in the figure. The initial reduction of the value of *R*_gyr_ corresponds to the extent of collapse of the inner bore, which is found to be complete in the high-temperature regime and incomplete in the low-temperature regime. The temperature-dependence of the time of collapse in the high-temperature regime is found to follow the Arrhenius relation (see inset).

A more direct measure of the kinetics of initial rapid collapse (stage 1) can be obtained by studying the time evolution of radius of gyration, which gives the estimate of the extent of collapse of the inner bore. For an *N*-atom simulation cell, the radius of gyration, *R*_gyr_, is defined as


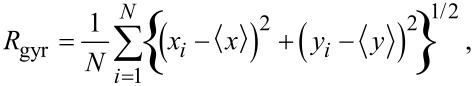


where *x_i_* *and y_i_* denote the lateral coordinates of the *i-*th atom, perpendicular of the axis of the wire, with <*x*> and <*y*> denoting the corresponding components of the center of mass. Figure 6 shows the time variation of *R*_gyr_ for the first 100 ps of the simulation. Collapse of the nanostructure is reflected in the drastic reduction in the value of *R*_gyr_ during this time. We find that the time of collapse of the hollow core is sensitive to the temperature and the rate of collapse decreases with decrease in temperature. For instance, the bore takes about twice as much time to collapse at 650 K than at 750 K temperature. A further decrease in temperature to 575 K, belonging to the low-temperature regime (*T* < *T*_cross_) characterized by the inhomogeneous evolution process along the length of the wire, causes the collapse to be five-fold slower. Focusing on the high-temperature regime with spatially uniform morphological evolution (*T* ≥ *T*_cross_), the time of collapse (*t*_col_) is found to follow Arrhenius behavior, 
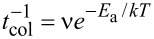
, *E*_a_ being an effective activation energy and ν being the attempt frequency, as shown in the inset of Figure 6. The non-linear fit to the data yields the effective attempt frequency as 5 × 10^12^ Hz, while the activation energy turns out to be about 0.27 eV. The observed Arrhenius behavior suggests that the thermally activated processes are operative during the collapse involving the nucleation of dislocations. The order of the obtained value of activation energy agrees well with that reported for the nucleation energy of dislocation [26], thereby providing support to this hypothesis.

### Atomic volume

Because the radius of gyration becomes constant at the end of stage 1, a different measure is required to analyze the subsequent stages of recrystallization and slow recovery. In this context, the atomic volume serves as an important measure. In this study, we restrict the analysis to the high-temperature regime in which different stages of evolution happen uniformly across the length of the NW.

**Figure 7:**
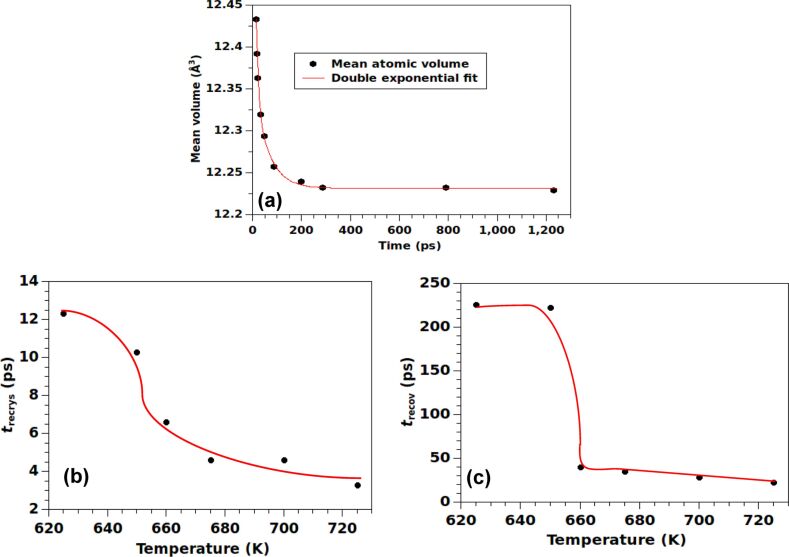
(a) Double exponential fit to the mean atomic volume of the simulated nanostructure as a function of time during the recrystallization and recovery processes in the high-temperature regime. A representative case for *T* = 700 K is given. The time constants corresponding to (b) recrystallization and (c) recovery are plotted as functions of temperature. The solid curves are to guide the eye.

The calculated atomic volume is defined as the volume available to an atom obtained by the Voronoi-meshing of the atomic configuration, implemented through the algorithm of division of space [27]. Mean value of the atomic volume is computed at different temperatures. The available volume of a disordered atom is larger than that of an atom in a crystal. Therefore, the recrystallization and recovery involving transformation of disordered atoms to crystalline atoms is associated with a drop in the mean atomic volume, as shown in the typical example of collapse at 700 K presented in Figure 7a. The calculated data points for temporal evolution of the atomic volume can be fitted by a double-exponential form for the decay of the mean atomic volume. The corresponding time constants, *t*_recrys_ and *t*_recov_ can be attributed to the time scales associated with stage 2 (recrystallization) and stage 3 (recovery), respectively. Both of these time constants exhibit a general decreasing trend upon increasing the temperature, with a jump at around 650 K (*T*_jump_), as shown in Figure 7b and Figure 7c. As explained before, the process of recrystallization and recovery involves the growth and subsequent elimination of stacking faults within the interior of the NW, after the inner surface is eliminated at the end of stage 1. We note that the nucleation of faults and defects is energetically less expensive in the presence of a surface, and that of an effective load generated by the surface tension. This presumably explains the fact that *T*_jump_ is somewhat higher than *T*_cross_, as the latter is dictated by the kinetics in the early stage of collapse, rather than by that of the late stage.

## Conclusion

In conclusion, we numerically investigate the thermal stability of hollow crystalline nanowire through molecular dynamics simulation. The results reveal that the hollow nanowire undergoes a rapid collapse, thereby transforming into a structure of reduced diameter consisting of disordered atoms and some residual crystalline parts with stacking faults. The crystalline parts act as nucleation centers for the recrystallization of the disordered atoms. This recrystallization also involves the growth of stacking faults. Finally, healing of the wire occurs through annihilation of the planar defects, leaving behind a single crystalline solid nanowire with few twin boundaries. We also study the kinetics of these processes, and compute the associated time scales. This work highlights the elasto-plastic mechanism of collapse of a hollow nanowire driven by plastic slips and the subsequent recrystallization through elimination of stacking faults. The suggested mechanism is different from the existing notion of diffusive transport of vacancies. We hope that the microscopic understanding gained in the present study will motivate further experimental investigations on the structural stability of hollow nanomaterials.
